# FT-Raman Methodology Applied to Study the Effect of Time and Type of Seasoning in the Crafting of *Sherry Casks^®^* Used in the Aging of Brandy De Jerez

**DOI:** 10.3390/s23218962

**Published:** 2023-11-03

**Authors:** María Guerrero-Chanivet, Dominico A. Guillén-Sánchez, Manuel José Valcárcel-Muñoz, M. Valme García-Moreno, Ofelia Anjos

**Affiliations:** 1 Departamento de Química Analítica, Facultad de Ciencias, Instituto de Investigación Vitivinícola y Agroalimentaria (IVAGRO), Campus Universitario de Puerto Real, Universidad de Cádiz, 11510 Puerto Real, Spain; maria.guerreroch@uca.es (M.G.-C.); dominico.guillen@uca.es (D.A.G.-S.); 2 Bodegas Fundador S.L.U., C/San Ildefonso, n 3, 11403 Jerez de la Frontera, Spain; mjc.valcarcel@gmail.com; 3 CERNAS-IPCB, Research Centre for Natural Resources, Environment and Society, Polytechnic Institute of Castelo Branco, 6001-909 Castelo Branco, Portugal; 4 Centro de Biotecnología de Plantas da Beira Interior, 6001-909 Castelo Branco, Portugal

**Keywords:** spectroscopy, wine spirit, Sherry wine, aging, chemometrics

## Abstract

Brandy de Jerez is a grape-derived spirit produced in Southern Spain with specific characteristics that come from the casks where it is produced, which must have previously contained some type of Sherry wine for at least 12 months. These casks are known as *Sherry Cask*^®^. In this work, Brandies de Jerez aged for different aging times (0, 3, 6 and 12 months) in casks seasoned with three different types of Sherry wines (Fino, Oloroso and Amontillado) have been studied. The samples have been analyzed using FT-Raman spectroscopy, and their chemical characterization has also been realized by studying their total content of organic acid, volatile compounds, and phenolic and furanic compounds. Their chemical study showed that the main differences between the studied samples were due to the duration and the type of seasoning performed. However, the spectra obtained through FT-Raman presented noticeable differences according to cask seasoning time and the Sherry wine used for the process. A PCA (Principal Component Analysis) confirmed that the Brandies de Jerez presented significant differences depending on the seasoning time and type that the casks were subjected to. A PLS-R (Partial Least Squares Regression) study enabled establishing a close correlation between specific regions of the FT-Raman spectra and cask seasoning time.

## 1. Introduction

Brandy de Jerez is a grape-derived spirit produced in the Southern Spanish area known as Marco de Jerez. The Technical File of the Protected Geographical Indication for Brandy de Jerez contains the specifications that must be observed for its production. This document defines Brandy de Jerez as a grape-derived spirit with a minimum alcoholic strength of 36% Alcohol by Volume (ABV) (normally between 36% and 45% ABV), obtained exclusively from wine spirits and distillates aged in under 1000 L oak casks which have been previously seasoned with Sherry wine (*Sherry Cask*^®^), following a traditional dynamic aging system employed in the Sherry area known as *Criaderas and Solera* [1,2]. Brandy de Jerez exhibits a number of specific organoleptic characteristics that make it different from other spirits [3]. Such characteristics are conferred to the brandy by the unique casks where they are aged. This is so because the casks used to produce Brandy de Jerez must have previously contained some type of Sherry wine, namely Fino, Oloroso, or Pedro Ximénez. This conditioning process is known as *seasoning* and has to be carried out following the indications established by the Technical File that regulates its elaboration [4]. These seasoned casks are then referred to as *Sherry Cask*^®^. During the Brandy de Jerez ageing process, the organoleptic characteristics of the spirit are largely influenced by numerous factors, such as the cask wood type, the temperature and humidity in the cellar, the ageing time and, only in the case of Brandy de Jerez, the seasoning of the casks [3]. There are other aspects of the cask that also affect the compounds that the wood transfers to the brandy, such as its botanical origin, the volume of the cask or the toasting treatment applied to the cask wood. Traditionally, American oak (*Quercus alba*), after a medium toasting treatment, is the wood most often employed for the manufacturing of Brandy de Jerez ageing casks.

In addition to typical wood compounds, *Sherry Casks*^®^ act like vectors that transfer certain compounds contributed by the seasoning wine into the distillates. This modulates the organoleptic characteristics of each particular brandy according on the type of Sherry wine used to season the brandy-ageing casks. Fino, Oloroso and Pedro Ximénez are some of Sherry wines most often used to condition *Sherry Casks*^®^. Sherry wines are produced following two different methods: biological or oxidative ageing. Grapes of the Palomino variety are used to elaborate the dry Sherry wines, such as Fino or Oloroso [5,6]. Pedro Ximénez grapes are the ones employed to produce naturally sweet Sherry wines, such as Pedro Ximénez [7]. Fino Sherry is a dry wine, obtained by biological ageing under the action of flor velum yeast, and it is characterized by its pale color as well as its dry with slightly acid flavor and sharp aromas with hints of almonds [6]. Oloroso Sherry wine is a fortified wine obtained by oxidation. It contains greater alcohol contents than Fino Sherry wines to ensure an exclusively oxidative ageing process. It develops a dark color, and its aroma evolves differently from that of Fino Sherry wine [5]. Pedro Ximénez Sherry wines are also obtained through an oxidative ageing process, but they are produced from Pedro Ximénez grapes. This is a naturally sweet fortified wine, rich in sugars, as a consequence of the raisining process applied to the Pedro Ximénez grapes. These wines are only partially fermented in order to preserve their original sweetness and to ensure ageing in an oxidative environment. This enhances its complexity, while an intense color and a dense appearance are acquired [7]. The sensory profiles and the aromatic composition of these Sherry wines are very different and the compounds that the wood will retain during the seasoning process will also be very different. As a consequence of that, the character that Brandies de Jerez acquire during their ageing in *Sherry Casks*^®^ will also be very different. The Technical File that regulates the conditioning of these unique casks has established that the seasoning Sherry wine must remain for at least one year inside the casks for these to be granted the *Sherry Cask*^®^ designation [4].

Nowadays, the use of spectroscopic techniques, such as NIR, FTIR-ATR or FT-Raman, in the food and beverages field is on the rise [8,9,10,11,12]. These techniques allow the simultaneous measurement of numerous compounds. They are not invasive, do not require sample treatment (or just a simple treatment), and are also more rapid and less costly than other traditional chromatographic techniques as well as being environmentally friendly. Thus, these are really interesting techniques for use in the field of agrifood. Vibrational spectroscopy, in particular, has been widely applied in recent years in combination with Multivariate Data Analysis for the analysis of alcoholic beverages and spirits.

FT-Raman spectroscopy has also been used to control the protected geographical indications of different wines [13], to determine the ethanol content in spirits [14,15], to monitor honey’s spirit distillation processes [16], to control the quality of tequila [17] or for the discrimination of fruit distillates [18]. Nevertheless, only one novel study on the application of this technique to identify the different ageing stages of wine spirit has been published [19]. The main advantage of FT-Raman spectroscopy compared to spectroscopic techniques such as NIR and FTIR-ATR is that the spectra are not influenced by the water content in the samples, making it very interesting for the study of alcoholic beverages.

Only a few studies have been published that relate these techniques to wines and wine spirits [12,14,20,21,22,23], while no references with regard to the application of FT-Raman to the study of Brandy de Jerez have been found. Two of the most important variables in Brandy de Jerez elaboration are the kind of Sherry wine employed to season the cask and the seasoning length of time. The minimum seasoning time required to produce a *Sherry Cask*^®^ has been determined empirically and there are no research studies to support that 1 year is the optimal seasoning time. There are no references in the bibliography related to the seasoning time, so we have considered it of interest to determine whether any real differences between a 1-year seasoning time and shorter seasoning periods can be confirmed. In the same line, it would also be interesting to corroborate any possible differences between the seasonings conducted using different types of Sherry wines and their influence on the final Brandies de Jerez. In the present work, the impact and behavior of these variables have been examined by means of FT-Raman spectroscopy in order to corroborate whether a successful discrimination between Brandies de Jerez produced under different conditions would be achievable.

An unsupervised pattern recognition chemometric study (Principal Component Analysis—PCA) has been used to evaluate the possible differences or similarities between the different samples under study. In addition, a Partial Least Squares Regression study has revealed a good correlation between certain areas in the brandies’ spectra and the length of time that the seasoning wine has been aged in the casks later on used to age the brandies.

## 2. Materials and Methods

### 2.1. Samples

The experiments were carried out in 500 L wooden casks made of American oak (*Quercus alba*) after a medium toasting treatment (Tonelerías Domecq, Jerez de la Frontera, Spain). For the seasoning of the casks, Fino (F), Oloroso (O) and Pedro Ximénez (PX) Sherry wines were used. The casks were seasoned for 3, 6 and 12 months. The wines remained in their corresponding casks over the whole seasoning period. All of the Sherry wines were supplied by Bodegas Fundador, S.L.U (Jerez de la Frontera, Spain).

Once the stipulated time for the cask to be seasoned was reached, all of the Sherry wines were extracted from the casks. In addition, the casks were left to drain upside down for 72 h to ensure a complete removal of the wine. Once the casks had been drained, they were filled with wine spirit, which was left to age for 12 months. The same wine spirit (from grapes of the Airén variety) was used for all the experiments.

The wine spirit used for the experiments had been obtained by column distillation at 77% Alcohol by Volume (ABV) and hydrated until 65% ABV with demineralized water before ageing, which is the common ageing alcoholic strength of wine spirits in the Sherry area. All the wine spirit employed was supplied by Bodegas Fundador, S.L.U.

A summary with the conditions under which the wine spirits were aged can be seen in Table 1. To be used as the reference for the regression studies, a new unseasoned cask was used to age the same wine spirit as in the rest of the seasoned casks. These would be the brandy obtained at 0 seasoning time. Each experience was performed using two different casks. All the experiments were carried out in the same cellar, at the facilities provided by Bodegas Fundador, S.L.U. Individual brandy samples were taken from each cask after 12 months of ageing.

### 2.2. Chemicals and Reagents

Ultrapure water (EMD Millipore, Bedford, MA, USA); UHPLC-grade acetone (VWR International, Radnor, PA, USA); and 0.1 M sulfuric acid (Sigma-Aldrich, Saint Louis, MO, USA) were used for the preparation of the eluent used for the analysis of organic acids.

For the analysis of phenolic and furanic compounds, HPLC-grade acetonitrile, supplied by Panreac (Barcelona, Spain); acetic acid, supplied by Merck (Darmstadt, Germany); and ultrapure water, supplied by EMD Millipore (Bedford, MA, USA) were used to prepare the UHPLC phases.

Sigma-Aldrich (Saint Louis, MO, USA) supplied all of the reagents for the analysis of the oenological control parameters in addition to the calibration standards.

### 2.3. Chemical Analysis

Organic acids (tartaric acid, malic acid, succinic acid, lactic acid, acetic acid) were analyzed by ion chromatography, according to the methodology described in other articles of the research group [6].

The aldehydes (acetaldehyde, acetaldehyde-diethylacetal), higher alcohols (2-propanol, n-propanol, hexanol, isobutanol, n-butanol, 2-phenylethanol, 2-methyl-1-butanol, 3-methyl-1-butanol), ethyl esters from fatty acids (ethyl hexanoate, ethyl octanoate, ethyl decanoate, ethyl dodecanoate, ethyl tetradecanoate, ethyl hexadecanoate) and ethyl esters from organic acid (ethyl acetate, ethyl lactate, diethyl succinate, diethyl malate, diethyl tartrate) content of each sample were analyzed by Gas Chromatography-Flame Ionization Detection (GC-FID) according to the methodology described in other articles of the research group [24].

Phenolic and furanic compounds (gallic acid, hydroxymethylfurfural, furfural, vanillic acid, 5-methylfurfural, syringic acid, vanillin, p-coumaric acid, syringaldehyde, coniferaldehyde, sinapaldehyde) were analyzed by Ultra-Performance Liquid Chromatography (UPLC) according to the methodology previously developed by our research group [25].

The samples were measured directly in quadruplicate. All the results were expressed in mg/L.

### 2.4. FT-Raman Spectra Acquisition

The spectral data on the Brandy de Jerez samples were obtained using a FT-Raman spectrometer BRUKER MultiRAM (Bruker Optik, Germany) according to the methodology used by Anjos et al. [16]. Briefly, 100 scans per spectrum at a spectral resolution of 8 cm^−1^, Raman laser power of 450 mW, with a scanner velocity of 5 kHz in the wavelength range from 3500 to 100 cm^−1^ were used. The FT-Raman spectrometer BRUKER MultiRAM was equipped with a 180° high-throughput collecting lens, a ultra-high-sensitivity Liquid Nitrogen cooled Ge Diode detector, an integrated 1064 nm, diode pumped, Nd:YAG laser with a maximum output power of 500 mW. The system was operated using the OPUS application software provided by the manufacturer. Four independent measurements were performed on each sample in a 5 mm optic space quartz cell with the opposite face mirrored. Each sample was analyzed in quadruplicate.

### 2.5. Chemometric Analysis

The software packages Statgraphics 19 (Statgraphics-Technologies, Inc., The Plains, VA, USA) was employed for the ANOVA and the Fisher’s Least Significant Difference test (chemical analysis) and Statistica 7.0 from Statsoft was used for the analytical Principal Component Analysis. The spectra obtained through FT-Raman are complex and, on some occasions, they may become even more complex because of external factors, such as sample heterogeneities, instrumental noise or scattering effects. For these reasons, appropriate chemometric tools, namely spectral pre-processes, are usually applied in order to improve the accuracy of the analyses. In this work, a variety of pre-processes were tested to determine the most appropriate one according to the data of interest: baseline correction, normalization, Multiplicative Scatter Correction (MSC), Standard Normal Variate (SNV), first and second derived Savitzky–Golay (1st derived, 2nd derived) as well as several combinations including 2 to 3 pre-treatments. Principal Component Analyses (PCA) of the pre-processed spectral data were used to determine any possible groupings or relations between the samples. These analyses were conducted by means of a Unscrambler X, version: 10.4 (CAMO Software AS, Oslo, Norway). For the spectral acquisitions and for their first evaluation, the software OPUS, version: 7.5.18 (Bruker Optik, Germany) was used. The Principal Component Analysis and the Partial Least Squares Regression were performed using Unscrambler X, version: 10.4 (CAMO Software AS, Oslo, Norway). The tables and plots were generated using Microsoft Excel 2016 (Microsoft Corp., Redmond, WA, USA).

## 3. Results

### 3.1. Analytical Characterization of the Brandies de Jerez

Table 2 shows the results of the physicochemical determinations performed in the brandies as well as the results of the Fisher’s Least Significant Difference test for each variable considered in each seasoning type.

The organic acids contents show significant differences depending on the seasoning time for each type of seasoning studied. The organic acid content of the brandies studied presents statistically significant differences depending on the seasoning length of time in which these brandies were aged. In all cases, the total organic acid content of the brandies is much higher in those aged in casks without season (C0). The total organic acid content includes acetic acid, which is mainly released by the wood [26], since it comes from the degradation of hemicellulose [27]. In non-seasoned casks, the availability of acetic acid is greater than in the other casks, where the wine has extracted part of the acetic acid during the seasoning process. This explains this difference between them. On the other hand, in the case of brandies aged in Fino *Sherry Casks*^®^ and Oloroso *Sherry Casks*^®^ with a seasoning period of 3–12 months, a higher total organic acid content was found in the casks with a longer seasoning period. In these *Sherry Casks*^®^, the acetic acid available in the wood is lower than in casks with 3 to 6 months of seasoning, but the contribution of the wine is greater. The wine contributes malic, tartaric, succinic and lactic acids, as well as acetic acid, which affects the total acid content in the brandies that subsequently aged in those casks [5,6,7]. The evolution of the total organic acids content in the brandies aged in Pedro Ximénez *Sherry Casks*^®^ is not so clear, since Pedro Ximénez is a very dense wine with a high sugar content that hinders its penetration into the wood: less extraction of acetic acid during the wrapping and less predictable behavior [7].

The behavior of aldehydes inaged brandies is similar in all the cases studied, with significant differences depending on the duration of seasoning. In all cases studied, the content of aldehydes in brandies aged in seasoned casks is higher than in brandies aged in non-seasoned casks (C0). Sherry wines provide these compounds [5,6,7], so the greater the amount of wine, the higher the total content of aldehydes in the brandies. The content of aldehydes is higher in the brandies aged in Fino *Sherry Casks*^®^ than in the others, because these compounds (acetaldehyde and diethyl-acetal) are involved in the metabolism of the flor yeasts [28].

The higher alcohols are very similar in all the cases studied, since the wine spirit used to elaborate these brandies was the same in all cases. This family of compounds is very much characterized by the wine used to produce the distillate and the distillation technique used [29,30,31]. Higher alcohols are not as affected by the seasoning process.

With respect to the family of esters derived from fatty acids, their contents hardly evolved in the analyzed brandies, so it can be concluded that they do not seem to be much affected by the seasoning process. However, the content of this family of compounds is slightly higher in brandies aged in *Sherry Casks*^®^ than in those aged in non-seasoned casks.

The evolution of esters derived from organic acids is not clear in all cases studied between 3 and 12 months of seasoning. Among these acids, on the one hand, ethyl acetate (derived from acetic acid, whose main source is wood) is considered, which decreases with increasing seasoning time. On the other hand, ethyl lactate, diethyl succinate, diethyl malate and diethyl tartrate are considered, derived from the corresponding organic acids, which will have higher levels in the brandies for longer periods of seasoning. These two opposing effects mean that the evolution of these compounds in relation to the duration of seasoning is not clear, although this variable clearly affects this family of compounds. Brandies aged in non-seasoned casks have the highest content of esters derived from organic acids, due to their high content of acetic acid.

The content of phenolic and furanic compounds is much higher in brandies aged in non-seasoned casks than in brandies aged in seasoned casks. These compounds come mainly from the wood [3,26,32]. In all the cases studied, the phenolic and furanic content of the samples decreases with the duration of seasoning, since the wines used to season the cask before the brandies are aged extract some of the phenolic and furanic compounds from the wood [33,34], making them less available for the brandies that later aged in them.

The longer the seasoning, the lower the availability of phenolic and furanic compounds in the wood and the lower the content in the already aged brandies. The content of phenolic and furanic compounds in content of the brandies aged in Pedro Ximénez *Sherry Casks*^®^ is higher than the respective ones aged in Fino and Oloroso *Sherry Casks*^®^, mainly due to the following two reasons: (i) the high density and hydrophobicity of Pedro Ximénez Sherry wine, which hinders the penetration of the wine into the wood pores during the seasoning period. This leads to a poorer extraction of polyphenols compared to dry wines [35,36]. This poorer extraction of polyphenols enables greater availability of phenolic and furanic compounds that can be transferred from the wood to the brandies aged in these casks. (ii) Pedro Ximénez Sherry wine is rich in furfural and its derivatives [7]. For this reason, it also contributes these compounds into the seasoned wood, which are later transferred to the brandies, making its total phenolic and furanic content higher than the others.

**Figure 1:**
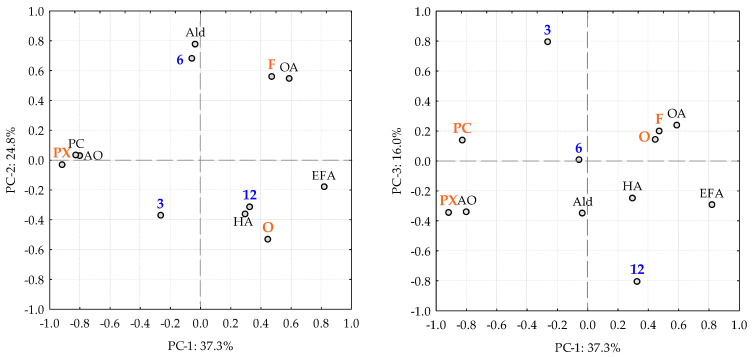
PCA representation of loadings and scores of analytical characterizations of brandies aged in different *Sherry Casks*^®^ considering the effect of the seasoning type and the seasoning time. F: Fino; O: Oloroso; PX: Pedro Ximénez. The number indicates the number of seasoning months. PC: phenolic compounds; OA: organic acids; Ald: aldehydes; EOA: esters derived from organic acids; EFA: esters derived from fatty acids; HA: higher alcohols.

In this case, the PCA explains 76.1% of the total variation (Figure 1) and the PC-1 that explains 37.3% of the variance explain the difference between PX (sweet Sherry wine) and dry Sherry wines (F and O). This PC is influenced positively (F and O) by esters derived from organic acids and fatty acids, and higher alcohols, while the negative part (PX) is influenced by phenolic and furanic compounds and organic acids. PC-2 explains 22.8% of the variance explain the difference between F and O. F samples are influenced by aldehydes and esters derived from organic acids, while O samples are most influenced by higher alcohols and esters derived from fatty acids. PC-3 explains the difference between the seasoning months. The parameters studied affect this component equally, without highlighting any of them.

The analytical compounds studied enables the differentiation between the brandies according to the seasoning time and the seasoning type of the casks where they were aged.

### 3.2. The FT-Raman Spectra of the Brandies de Jerez

**Figure 2:**
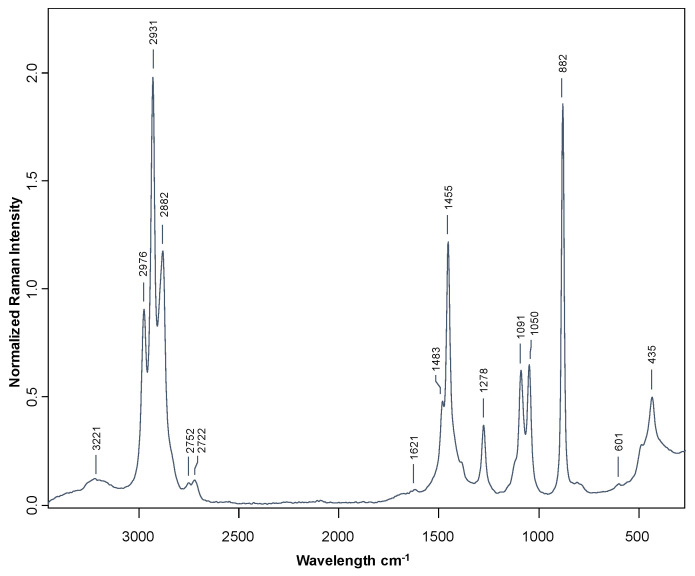
FTRaman spectra of a representative Brandy de Jerez sample.

The average FT-Raman spectra of a representative Brandy de Jerez is plotted in Figure 2, where the spectral information recorded is in accordance with previous reports by other authors [16,19].

The most relevant bands in the FT-Raman are marked in Figure 2 and the information related with the interpretation of the most significant peaks are included in Table 3.

**Figure 3:**
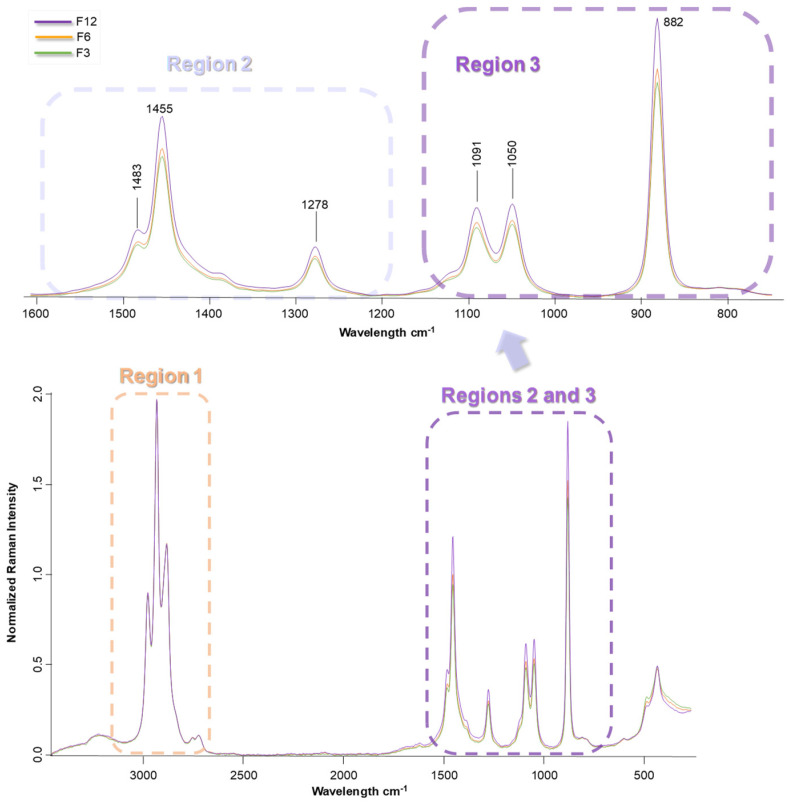
Most relevant regions of FT-Raman spectra of a representative Brandy de Jerez aged in Fino *Sherry Casks*^®^ samples. F: Fino. The number after the code of Sherry wine indicates the number of seasoning months.

Figure 3 highlights the relevant regions in the FT-Raman spectra. This plot corresponds to the Brandies de Jerez aged in Fino *Sherry Cask*^®^, although all the brandies that have been studied presented very similar behaviors. Thus, just some minor differences can be observed in region 1 highlighted in the spectrum. However, it can be seen in Figure 3, that regions 2 and 3 contain the most relevant data differences for further analysis.

PCA with mean centering and a cross-validation method were performed and used to discriminate between the different Brandy de Jerez samples based on their qualitative analysis. The first derivative with 17 smooth values was the pre-process that provided the most relevant information. Thus, in the first place, the PCA was performed using the whole spectra information (from 3510 cm^−1^ to 310 cm^−1^) from the groups of brandies aged in each type of *Sherry Casks*^®^ in order to determine if the 1st derivative in regions 2 and 3 of the average FT-Raman spectra were the most relevant regions for the successful discrimination of the Brandies de Jerez.

**Figure 4:**
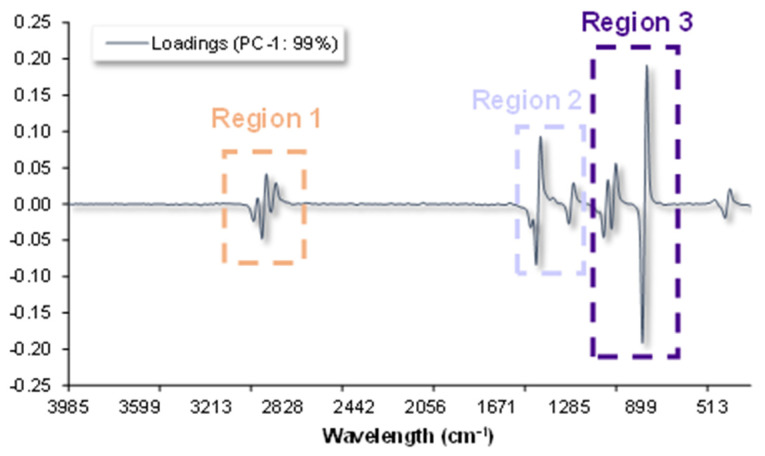
Loadings in PC-1 of Brandy de Jerez aged in Fino *Sherry Casks*^®^ PCA performed with the 1st derivative of the spectral data.

The loadings in PC-1 according to the PCA model applied to the Brandy de Jerez samples that had been aged in Fino *Sherry Cask*^®^ are shown in Figure 4. The regions 1, 2 and 3 seem to be the most relevant with regard to the grouping of the samples. Region 1 comprises the wavelength range from 3090 cm^−1^ to 2770 cm^−1^. The bands that appear in this region are associated with –H and –OH stretching modes (highly influenced by methanol and ethanol content). Region 2, that corresponds to the wavelengths 1610 cm^−1^ to 1215 cm^−1^, comprises the bands attributable to –CH_2_, –CH_3_ bending and H-C-H bending modes. Region 3, from 1170 cm^−1^ to 785 cm^−1^ wavelengths displays the effect of C-O stretching vibration, –CH_3_ rocking vibrations and C-C stretching (Table 3). The most relevant regions observed in this PCA model were the same for all the different Brandies de Jerez studied.

A separate PCA was performed on each region to determine which region or combination of them was the most appropriate to attempt a successful discrimination between samples. All the possible region combinations were tested. The best results were obtained when the PCA was performed on a combination of region 2 and region 3. The different profiles exhibited by each one of the tested brandies (Figure 3) suggested that these regions would be the most reliable reference to differentiate between samples through subsequent PCA analysis.

Region 1 corresponds to the spectra at approximately 3000 cm^−1^ wavelengths, which is strongly marked by the ethanol and methanol content in the samples. Since all the experiments were started using the same wine spirit and they were all aged at the same alcoholic strength, 65% ABV, the bands that appeared in this region of the spectra were rather similar. This rendered region 1 unsuitable to discriminate between sample types.

Region 2 was also shaped by the samples’ ethanol content, but the peak that appears at approximately 1200 cm^−1^ was also strongly influenced by the alcohols, ethers, esters, and carboxylic acids that originated from the seasoning of the casks. This made region 2 a suitable one to discriminate between the Brandy de Jerez seasoned with different Sherry wines under study.

Region 3 proved to be the most significant zone of the spectra for the successful discrimination of the samples. A strong Raman band, at approximately 800 cm^−1^, which corresponds to the primary and secondary alcohols present in the samples appeared in this region. The other bands in this region appeared at approximately 1000 cm^−1^ and were attributed to C-O vibrations. These C-O vibrations could come from carboxylic acids, which are some of the most characteristic and distinctive components that can be found in Brandies de Jerez. The presence of these organic acids and their derivative could only be explained by the corresponding wine contributions, which takes place during the seasoning of the casks. Thus, depending on the type of Sherry wine used for the cask seasoning (Fino, Oloroso, Pedro Ximénez) and the length of time that each cask holds that wine, a varying amount of phenolic and furanic compounds and organic acids would be transferred onto the wood and, in turn, into the final Brandy de Jerez. All these compounds have bonds that can be reflected in region 3 of the spectra, which makes of it the most relevant wavelength range when it comes to discriminate between Brandies de Jerez according to their seasoning length of time.

**Figure 5:**
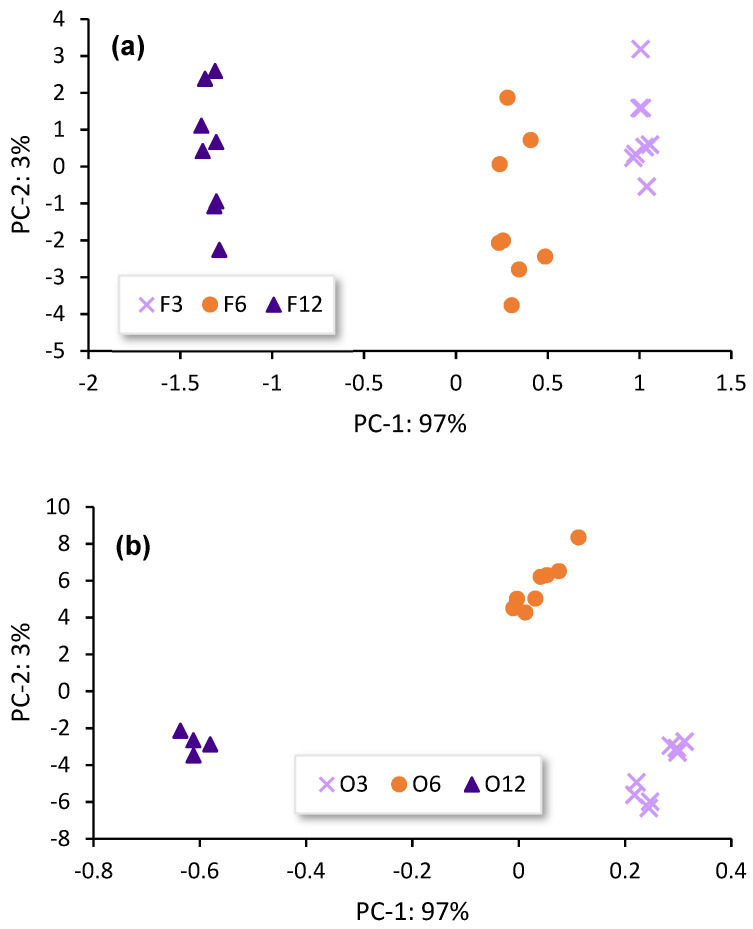

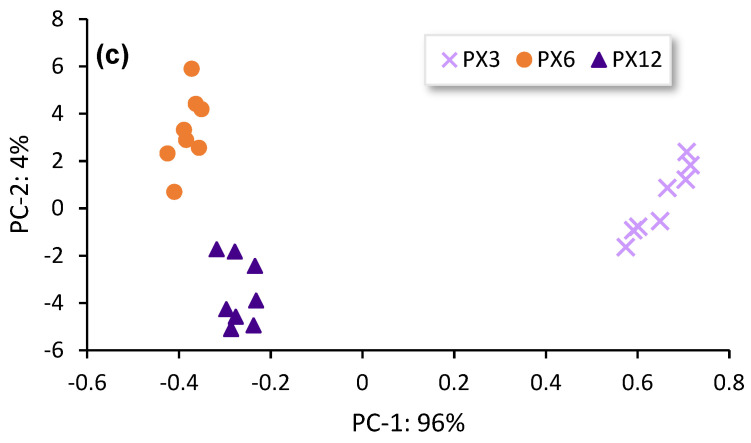
Scatter plot PC–2 vs. PC–1 of: (**a**) Brandies de Jerez aged in Fino *Sherry Casks*^®^; (**b**) Brandies de Jerez aged in Oloroso *Sherry Casks*^®^; and (**c**) Brandies de Jerez aged in Pedro Ximénez *Sherry Casks*^®^. F: Fino; O: Oloroso; PX: Pedro Ximénez. The number after the code of Sherry wine indicates the number of seasoning months.

A PCA for each type of cask-seasoning Sherry wine (Fino, Oloroso and Pedro Ximénez) was performed, following the same procedure above describe. On this occasion, only the spectral data from regions 2 and 3 were considered. Two principal components (PC) that explain more than 96% of the total variance of the system in all the cases studied were determined. In Table 4, the scores obtained through each model are shown. All the models explained at least 96% of the variance of the samples. Figure 5 shows the distribution of the Brandy de Jerez samples in the plane formed by PC-1 and PC-2 for each type of Sherry wine used to season the casks, and for the control experience. In all the experiences studied, the Brandies de Jerez formed different groups according to the length of time their *Sherry Casks*^®^ had been seasoned.

As can be observed in Figure 5, the groups of brandies aged in the *Sherry Casks*^®^ that had been seasoned using dry Sherry wines (Fino and Oloroso) follow the same pattern: PC-1 seems to be related with the seasoning time of the *Sherry Casks*^®^. The positive zone of PC-1 includes the brandies aged in the casks that had been seasoned for a shorter time, while the negative zone of the PC-1 contains those groups of the brandies aged in casks seasoned for 12 months. The longer the wine stayed in the casks, the more compounds were retained in the wood and the more substances were later on precipitated, so that the wine spirits aged in these casks would become richer in this type of compounds. As above explained, the bands that appeared in regions 2 and 3 of the FT-Raman spectra could be indicative of the compounds that had been contributed with by the seasoning wine, among other compounds. This explains why the brandies in PC-1 formed different groups according to their concentration of compounds from the Sherry wines, which, in turn, depended on the length of time that their corresponding casks had been seasoned. It is noteworthy that the pattern corresponding to the Brandies de Jerez aged in Pedro Ximénez-seasoned *Sherry Casks*^®^ is slightly different. This could be explained by the rather high density of this wine, because of its sugar content, that prevents it from penetrating into the wood pores to the same extent as the other wines. Further, the greater sugar content of these Brandies de Jerez may affect the C-O bonds, which would be reflected in the spectral bands that appeared in regions 2 and 3. Although the Brandies de Jerez aged in Pedro Ximénez-seasoned *Sherry Cask*^®^ do not follow the same pattern as the rest of the brandies, they still form groups that allow their discrimination.

The spectral profiles obtained through FT-Raman enabled differentiating between the different seasoning times of all the Brandies de Jerez studied, as they formed different groups according to their seasoning length of time.

**Figure 6:**
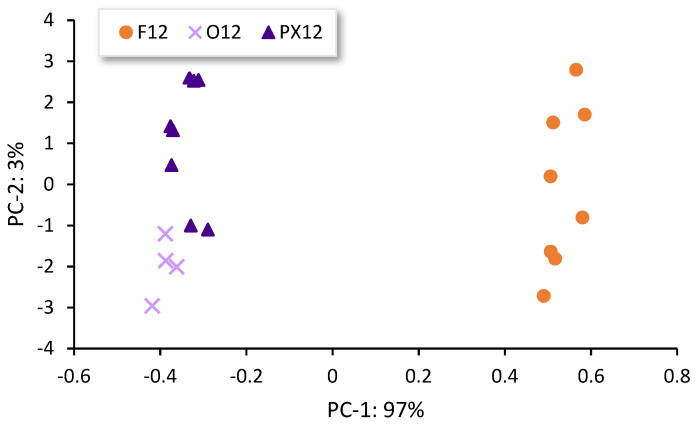
Scatter plot PC-2 vs. PC-1 of Brandies de Jerez aged in 12-month *Sherry Casks*^®^. F: Fino; O: Oloroso; PX: Pedro Ximénez.

Also, an invariable seasoning time was to be established in order to compare the behavior of the different Brandies de Jerez studied. Therefore, a seasoning time of 12 months was chosen, as this is the minimum time established by the Technical File of the Protected Geographical Indication [4]. As previously described, a PCA was performed on the spectral data from regions 2 and 3 of the Brandies de Jerez aged in 12-month seasoned casks. The first derivative with 17 smooth values was the pre-processing that provided the most relevant information. Two principal components were extracted from this PCA: PC-1 explained 97% of the total variance of the system and PC-2 explained 3% of it. As can be seen in Figure 6, two main groups of samples can be distinguished according to the type of Sherry-seasoning wine.

Thus, the group of samples formed by Brandy de Jerez aged in either Oloroso or Pedro Ximénez *Sherry Cask*^®^ were located in the negative zone of the PC-1. Both of these Sherry wines are produced under oxidative ageing, and they exhibit very different characteristics from those corresponding to wines produced through biological ageing. The second group that could be observed was located in the positive zone of PC-1 and was formed by Brandies de Jerez aged in Fino *Sherry Cask*^®^ (biological ageing). This component (PC-1) seems to be related to the oxidative ageing of the Sherry wine used to season the casks where the Brandy de Jerez is later on produced. Generally, wines produced by oxidative ageing can contribute with a greater variety of aroma and flavor compounds to the wood than those obtained by biological ageing. Thus, the casks seasoned with Fino *Sherry Casks*^®^ can contain compounds derived from the yeast metabolism, these enrich the brandies with sharper aromatic notes. Based on the data obtained from this study, we can conclude that the type of Sherry wine used to season the *Sherry Casks*^®^ is a crucial factor with regard to the Brandies de Jerez which can be discriminated according to the different FT-Raman spectral profiles obtained for each type of seasoning Sherry wine.

The natural groupings observed in the Principal Component Analysis carried out with the first derivative of regions 2 and 3 of the FT-Raman spectrum agree with those observed in the Principal Components Analysis carried out with the analytical data in Section 3.1, adding value to the use of this technique in the differentiation of brandies in terms of time and type of seasoning of the casks where they were aged.

### 3.3. The Partial Least Squares Regression Model

Since the unsupervised pattern recognition analysis (PCA) revealed the clustering of the different brandy samples according to the seasoning length of time of the casks, a regression study through PLS-R should allow us to correlate such clusters with the most significant regions (regions 2 and 3) in the FT-Raman spectra and this would let us discriminate the brandies according to the length of time that their casks had been previously seasoned.

A model was constructed for each type of Sherry-seasoning wine studied (Fino, Oloroso and Pedro Ximénez). In all the cases, the signals from a reference Brandy aged for 12 months in new, unseasoned wood was considered as time 0. The studies were based on the first derivative of the signal, since, after testing different pre-processing methods, it proved to be the one to produce the best results. The data were mean centered to correct any possible heterogeneities and a cross-validation was performed. All the models were constructed according to PLS Kernel algorithm. The dimensions of the three data matrices studied were 32 × 407.

**Figure 7:**
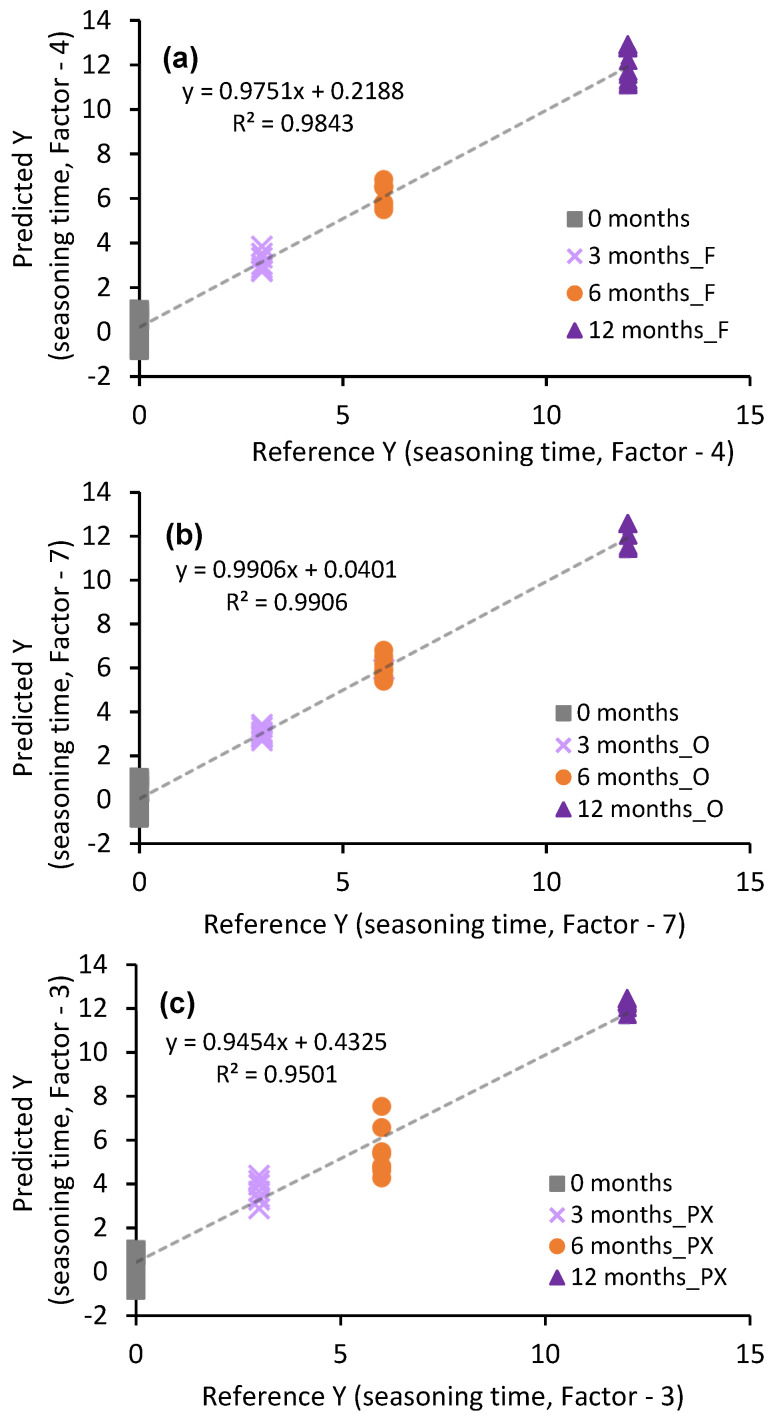
Regression line drawn against the prediction obtained in the calibration model using PLS (**a**) Brandies de Jerez aged in Fino *Sherry Casks*^®^, (**b**) Brandies de Jerez aged in Oloroso *Sherry Casks*^®^ and (**c**) Brandies de Jerez aged in Pedro Ximénez *Sherry Casks*^®^.

The results obtained are shown in Figure 7a–c. In the case of Brandies de Jerez aged in casks seasoned with Fino Sherry wine (Figure 7a), the regression coefficient was 0.9843 and the value of the slope between the estimated age and the actual measured age was 0.9843 in the model with 4 PCs. Regarding the Brandies de Jerez aged in Oloroso Sherry wine casks (Figure 7b), the regression coefficient was 0.9906 and the value of the slope between the estimated age and the actual measured age was 0.9906 in the model with 7 PCs. In the case of Brandies de Jerez aged in casks seasoned with Pedro Ximénez Sherry wine (Figure 7a), the regression coefficient was 0.9454 and the value of the slope between the estimated age and the actual measured age was 0.9454 in the model with three PCs.

Table 5 shows all the quality parameters of the three models developed. The three models were robust, with slopes and R^2^ coefficients close to 1 in all the cases, offsets close to 0 and low RMSECs (Root-Mean-Standard Error for Calibration) and RMSEP (Root-Mean-Squared Error of Prediction), which confirms the validity of the models. Table 5 also shows various statistical parameters associated to the equation that confirm the robustness of the models: graphic of predicted vs. reference (seasoning time). In all three cases, we found, as already mentioned, slopes must contain 1 and offsets must contain 0. The deviation associated to both, slope and offset, was low in all three models. The %P was below 95 in the three models, with the lowest value corresponding to the brandies aged in Oloroso *Sherry Casks*^®^. The model obtained from the samples aged in casks seasoned with Pedro Ximénez Sherry wine was the one to present the largest error, since it was also below 95%. The confidence interval of the three models includes 1 in the slope and 0 in the offset.

In addition, Table 6 shows, for each model, the data corresponding to the actual and the ageing time predicted by the model as well as the standard error (STD) of the prediction in all the cases studied. The model with the smallest cask seasoning-time prediction error was that of the Oloroso casks (STD error 0.399–0.425). On the other hand, the model that presented the greatest error was the one for Pedro Ximénez-seasoned casks, which did not exceed 1.263 in any of the cases. It should be noted that the brandies aged in Pedro Ximénez *Sherry Casks*^®^ have greater sugar contents than the rest, which may affect the FT-Raman measurements, since the C-O bonds present in the sugars affect the bands that appear in regions 2 and 3 of the spectra, which may result in a greater dispersion of the measurements.

The models built using the most relevant regions for the discrimination of the brandies according to the FT-Raman spectra have demonstrated to provide very good results. This represents a very useful tool for brandy producers, as they should be able to perform rapid and reliable measurements to determine the seasoning length of time of the casks where the brandy had aged. Further, this is a very interest study since the Technical Regulation for Brandy de Jerez establishes specific demands with regard to the elaboration of the brandies. These include the measurement of certain compounds content, such as tartaric acid, which must be detectable in the aged spirits and whose origin is attributed to the cask-seasoning wine. This acid is usually determined by liquid chromatography, and this measurement procedure may require a considerable length of time. In contrast, the spectrum of a brandy sample by FT-Raman can be obtained in just a few seconds. Given the close correlation between the FT-Raman spectrum of a particular brandy with the seasoning length of time in the cask where it aged, this measurement method represents a considerable improvement in terms of quality control efficiency.

## 4. Conclusions

It could be concluded that FT-Raman represents a promising technique to discriminate brandies based on both the seasoning time of the casks intended to be used for brandy ageing and the type of Sherry wine used for such seasoning. By performing the 1st derivative on the spectral data collected from each sample, good results were obtained. Nevertheless, only the spectral regions 1610 cm^−1^ to 1215 cm^−1^, and 1170 cm^−1^ to 785 cm^−1^ allowed the effective discrimination of the Brandies de Jerez according to the Sherry wine type used for the seasoning of their ageing casks or to the length of time that the seasoning was applied to these casks. The spectral bands that appear in these regions seem to be related to the seasoning Sherry wine compounds, which are transferred to the casks and, in turn, to the ageing brandies. In addition, the PLS-R models have been confirmed to accurately indicate the seasoning length of time of the casks where the brandies had aged according to the most relevant FT-Raman spectral regions. This would represent a considerable time-saving procedure to determine the ageing time of the casks in comparison to the usual analysis of samples. Brandy producers could benefit from a technique that enables determining the precise seasoning length of time of the cask determinations in just a few seconds.

The good results obtained through FT-Raman make this a highly interesting method for quality control purposes. It is, definitely, an inexpensive, non-destructive and time-saving technique that could be effectively used either by producers for the monitoring of their product quality or by regulatory entities for the prevention of fraud.

## Figures and Tables

**Table 1:** Experimental conditions under study.

Experience	Sherry Wine	Seasoning Time (Months)	Ageing Time (Months)
0 months	None	0	12
3 months	F, O, PX	3	12
6 months	F, O, PX	6	12
12 months	F, O, PX	12	12

F: Fino; O: Oloroso; PX: Pedro Ximénez.

**Table 2:** Analytical characterization of brandies aged in different *Sherry Casks^®^*.

Samples	Organic Acids (mg/L)	Aldehydes (mg/L)	Higher Alcohols (mg/L)	Esters from Fatty Acids (mg/L)	Esters from Organic Acids (mg/L)	Phenolic and Furanic Compounds (mg/L)
Fino *Sherry Casks*^®^						
C0	380.92 ± 21.24 ^c^	126.97 ± 2.08 ^a^	2039.28 ± 14.45	20.06 ± 0.15 ^a^	342.23 ± 7.39 ^b^	105.28 ± 25.44 ^b^
F3	200.71 ± 9.96 ^a^	139.74 ± 8.23 ^b^	2032.16 ± 55.17	25.73 ± 0.16 ^b^	308.51 ± 18.32 ^a^	38.73 ± 13.12 ^a^
F6	216.21 ± 28.61 ^ab^	147.53 ± 9.56 ^c^	2025.89 ± 18.53	26.22 ± 0.75 ^c^	311.94 ± 17.99 ^a^	39.10 ± 20.64 ^a^
F12	225.24 ± 1.93 ^b^	149.51 ± 4.75 ^c^	2054.50 ± 2.54	29.23 ± 0.23 ^d^	306.99 ± 11.11 ^a^	26.93 ± 6.58 ^a^
Oloroso *Sherry Casks*^®^						
C0	380.92 ± 21.24 ^b^	126.97 ± 2.08 ^a^	2039.28 ± 14.45 ^ab^	20.06 ± 0.15 ^a^	342.23 ± 7.39 ^c^	105.28 ± 25.44 ^a^
O3	213.50 ± 35.85 ^a^	124.12 ± 14.21 ^a^	2044.19 ± 1.77 ^ab^	26.27 ± 1.30 ^b^	281.12 ± 2.82 ^a^	48.41 ± 13.35 ^ab^
O6	204.66 ± 31.64 ^a^	143.11 ± 11.70 ^b^	2031.24 ± 7.94 ^a^	29.73 ± 0.29 ^c^	305.12 ± 20.14 ^b^	45.85 ± 12.84 ^b^
O12	216.99 ± 16.05 ^a^	137.05 ± 2.67 ^b^	2051.92 ± 20.07 ^b^	30.14 ± 0.84 ^c^	291.33 ± 5.00 ^a^	30.18 ± 16.01 ^c^
Pedro Ximénez *Sherry Casks^®^*						
C0	380.92 ± 21.24 ^c^	126.97 ± 2.08 ^a^	2039.28 ± 14.45 ^ab^	20.06 ± 0.15 ^a^	342.23 ± 7.39 ^c^	105.28 ± 25.44 ^c^
PX3	271.22 ± 3.40 ^b^	138.46 ± 9.43 ^b^	2028.32 ± 54.99 ^ab^	23.49 ± 0.20 ^b^	273.07 ± 15.14 ^b^	76.49 ± 3.67 ^b^
PX6	271.94 ± 5.17 ^b^	154.58 ± 1.69 ^c^	2012.87 ± 23.37 ^a^	23.47 ± 0.16 ^b^	276.73 ± 4.03 ^b^	68.28 ± 6.09 ^b^
PX12	254.17 ± 5.18 ^a^	138.32 ± 4.87 ^b^	2045.91 ± 18.62 ^b^	26.05 ± 0.23 ^c^	262.72 ± 5.08 ^a^	48.28 ± 1.23 ^a^

The results were expressed as the mean ± standard deviation (*n* = 4). C: control; F: Fino; O: Oloroso; PX: Pedro Ximénez. The number after the code of Sherry wine indicates the number of seasoning months ANOVA: different letters indicate significant differences (*p* < 0.05).

**Table 3:** Most relevant bands in FT-Raman Brandy de Jerez spectra interpretation.

Wavelength (cm^−1^)	Interpretation
3000–3500	–OH stretching [21]
2700–3000	–H and –OH stretching modes (ethanol, methanol, hydrocarbons, and other organic molecules) [37,38]
1480–1450	–CH_2_, –CH_3_ bending (also influenced by ethanol content in the samples) and H-C-H bending modes [21,39]
1278	H-C-C stretching [39]
1091–1050	C-O stretching vibration (associated with the presence of ethanol and methanol content), CH_3_ rocking vibrations and C-C stretching [37]
882	C-C stretching vibration (is the most characteristic band of ethanol) [38]
430–490	O-C-C bending [40]

**Table 4:** Principal Component extracted in PCA analysis performed with region 2 and region 3 of the FT-Raman spectra.

Model	% Variance Explain	
	in PC-1	in PC-2 (%)
Fino (F)	97	3
Oloroso (O)	97	3
Pedro Ximénez (PX)	96	4

**Table 5:** Statistical parameters of the obtained PLS-R model equations for brandies aged in Fino *Sherry Casks*^®^, for brandies aged in Oloroso *Sherry Casks*^®^ and for brandies aged in Pedro Ximénez *Sherry Casks*^®^.

	Fino (F)		Oloroso (O)		Pedro Ximénez (PX)	
Statistic of regression equation						
R^2^ (Pearson)	0.9751		0.9906		0.9454	
RMSEC	0.6483		0.3754		1.1224	
RMSEP	1.8593		0.3755		2.1751	
Statistic of slope and offset						
	*Slope*	*Offset*	*Slope*	*Offset*	*Slope*	*Offset*
Value	0.9751	0.2188	0.9906	0.0401	0.9454	0.4325
Standard deviation	0.0264	0.1814	0.0189	0.1092	0.0447	0.3071
*p* value	0.423	0.541	0.624	0.716	0.162	0.282
% *p*	57.7	45.9	37.6	28.4	83.8	71.8
t (0.05,32-2)	0.812	0.618	0.496	0.368	1.434	1.096
Lower limit (95%)	0.9572	−0.0001	0.9813	−0.0001	0.8719	−0.0005
Upper limit (95%)	1.0001	0.2243	1.00001	0.0803	1.0001	0.6724

**Table 6:** Prediction of the seasoning time of the casks where the samples had aged using the PLS-R model.

	ST	ST Predicted	STD Error
Brandy de Jerez aged in Fino *Sherry Casks^®^*			
C0	0	0.139	0.702
F3	3	3.240	0.690
F6	6	6.143	0.688
F12	12	11.853	0.711
Brandy de Jerez aged in Oloroso *Sherry Casks^®^*			
C0	0	0.027	0.407
O3	3	3.028	0.399
O6	6	5.986	0.400
O12	12	11.918	0.425
Brandy de Jerez aged in Pedro Ximénez *Sherry Casks^®^*			
C0	0	0.483	1.242
PX3	3	3.786	1.222
PX6	6	5.445	1.218
PX12	12	11.786	1.263

ST: seasoning time (months); C: control (without seasoning), F: Fino, O: Oloroso, PX: Pedro Ximénez. The number after the Sherry wine code indicates the seasoning time in months.

## Data Availability

Not applicable.
